# Bis(2-amino­benzonitrile)tetra­aqua­cobalt(II) dichloride

**DOI:** 10.1107/S1600536809050272

**Published:** 2009-11-28

**Authors:** Zong-Ling Ru, Guo-Xi Wang

**Affiliations:** a Department of Chemical & Environmental Engineering, Anyang Institute of Technology, Anyang 455000, People’s Republic of China

## Abstract

In the crystal structure of the title compound, [Co(C_7_H_6_N_2_)_2_(H_2_O)_4_]Cl_2_, the Co^II^ cation lies on an inversion center and is coordinated by two 2-amino­benzonitrile ligands and four water mol­ecules in a distorted octa­hedral geometry. The Cl^−^ counter-anion links with the complex cations *via* O—H⋯Cl and N—H⋯Cl hydrogen bonding. Inter­molecular O—H⋯N hydrogen bonding links the complex cations, forming supra­molecular chains running along the *b* axis.

## Related literature

For the chemistry of nitrile derivatives, see: Jin *et al.* (1994 ▶); Brewis *et al.* (2003 ▶). For a related structure, see: Fu & Zhao (2007 ▶).
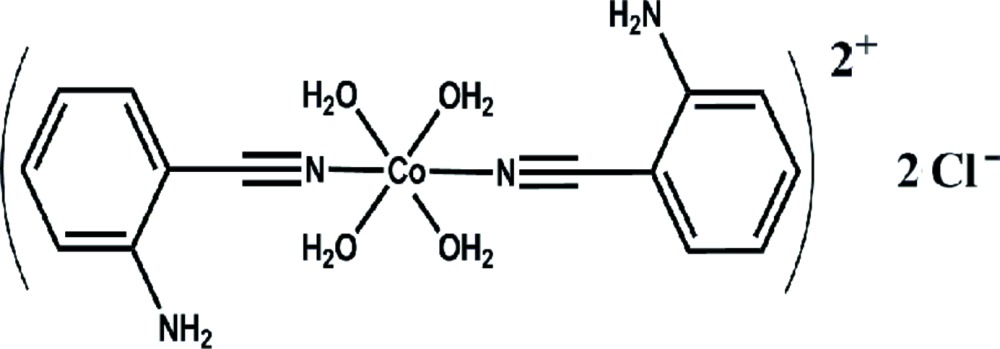


## Experimental

### 

#### Crystal data

[Co(C_7_H_6_N_2_)_2_(H_2_O)_4_]Cl_2_
*M* *_r_* = 438.17
Monoclinic, 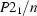

*a* = 12.492 (3) Å
*b* = 6.5864 (13) Å
*c* = 12.608 (3) Å
β = 109.24 (3)°
*V* = 979.4 (3) Å^3^
*Z* = 2
Mo *K*α radiation
μ = 1.17 mm^−1^
*T* = 298 K
0.35 × 0.30 × 0.15 mm

#### Data collection

Rigaku Mercury2 diffractometer
Absorption correction: multi-scan (*CrystalClear*; Rigaku, 2005 ▶) *T*_min_ = 0.732, *T*_max_ = 0.871
9255 measured reflections
2227 independent reflections
1872 reflections with *I* > 2σ(*I*)
*R*_int_ = 0.038

#### Refinement

*R*[*F* ^2^ > 2σ(*F* ^2^)] = 0.034
*wR*(*F* ^2^) = 0.072
*S* = 1.13
2227 reflections
115 parameters
4 restraints
H-atom parameters constrained
Δρ_max_ = 0.30 e Å^−3^
Δρ_min_ = −0.35 e Å^−3^

### 

Data collection: *CrystalClear* (Rigaku, 2005 ▶); cell refinement: *CrystalClear*; data reduction: *CrystalClear*; program(s) used to solve structure: *SHELXTL* (Sheldrick, 2008 ▶); program(s) used to refine structure: *SHELXTL*; molecular graphics: *SHELXTL*; software used to prepare material for publication: *SHELXTL*.

## Supplementary Material

Crystal structure: contains datablocks I, global. DOI: 10.1107/S1600536809050272/xu2671sup1.cif

Structure factors: contains datablocks I. DOI: 10.1107/S1600536809050272/xu2671Isup2.hkl

Additional supplementary materials:  crystallographic information; 3D view; checkCIF report

## Figures and Tables

**Table 1:** Selected bond lengths (Å)

Co1—O1*W*	2.0899 (14)
Co1—O2*W*	2.0550 (13)
Co1—N1	2.1566 (15)

**Table 2:** Hydrogen-bond geometry (Å, °)

*D*—H⋯*A*	*D*—H	H⋯*A*	*D*⋯*A*	*D*—H⋯*A*
O1*W*—H1*WA*⋯Cl1^i^	0.84	2.33	3.1600 (16)	170
O1*W*—H1*WB*⋯Cl1^ii^	0.86	2.27	3.1099 (15)	167
O2*W*—H2*WA*⋯N2^iii^	0.91	1.99	2.868 (2)	162
O2*W*—H2*WB*⋯Cl1^iv^	0.88	2.27	3.1438 (17)	178
N2—H2*B*⋯Cl1^v^	0.92	2.53	3.4433 (18)	172

Symmetry codes: (i) 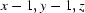; (ii) 
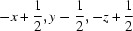
; (iii) 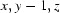; (iv) 
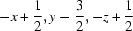
; (v) 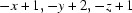.

**Fig. 1.**
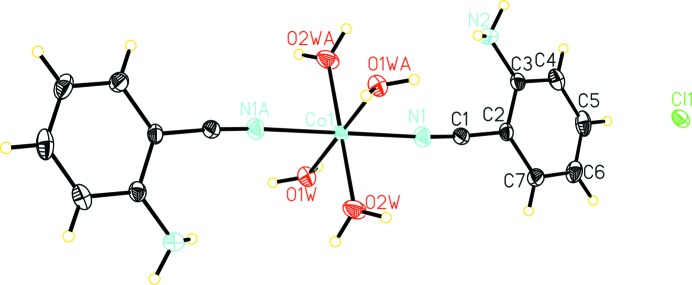
A view of the title compound with the atomic numbering scheme. Displacement ellipsoids were drawn at the 30% probability level.

## supplementary crystallographic information

### Comment

Nitrile derivatives have found wide range of applications in industry and coordination chemistry as ligands. For example, phthalonitriles have been used as starting materials for phthalocyanines (Jin *et al.*, 1994), which are important components for dyes, pigments, gas sensors, optical limiters and liquid crystals, and which are also used in medicine, as singlet oxygen photosensitisers for photodynamic therapy (Brewis *et al.*, 2003). Recently, we have reported a few benzonitrile compounds (Fu & Zhao, 2007). As an extension of our work on the structural characterization, we report here the crystal structure of the title compound tetra-aqua-bis(2-aminobenzonitrile)-cobalt(II) dichloride.

The crystal data show that in the title compound, the Co(II) lies on an inversion center. The distorted octahedral Co(II) environment contains two N atoms from two planar *trans*-related 2-aminobenzonitrile ligands in the axial positions and four aqua O atoms in the equatorial plane. In the crystal, O—H···Cl, N—H···Cl and O—H···N hydrogen bonds generate an infinite two-dimensional network (Fig.1).

### Experimental

A mixture of 2-aminobenzonitrile (0.1 mmol) and CoCl_2_ (0.1 mmol) and water (1 ml) sealed in a glass tube were maintained at 343 K. Crystals suitable for X-ray analysis were obtained after 5 d.

### Refinement

H atoms attached to C atoms were located geometrically and treated as riding with C—H = 0.93 Å, *U*_iso_(H) = 1.2*U*_eq_(C). H atoms bonded to O and N atoms were located in a difference Fourier map and refined with distance restraints of O—H = 0.85±0.03 and N—H = 0.89±0.03 Å, *U*_iso_(H) = 1.5*U*_eq_(O,N).

### Crystal data

**Table:**

[Co(C_7_H_6_N_2_)_2_(H_2_O)_4_]Cl_2_	*F*(000) = 450
*M**_r_* = 438.17	*D*_x_ = 1.486 Mg m^−^^3^
Monoclinic, *P*2_1_/*n*	Mo *K*α radiation, λ = 0.71073 Å
Hall symbol: -P 2yn	Cell parameters from 1872 reflections
*a* = 12.492 (3) Å	θ = 3.4–27.5°
*b* = 6.5864 (13) Å	µ = 1.17 mm^−^^1^
*c* = 12.608 (3) Å	*T* = 298 K
β = 109.24 (3)°	Block, red
*V* = 979.4 (3) Å^3^	0.35 × 0.30 × 0.15 mm
*Z* = 2	

### Data collection

**Table:**

Rigaku Mercury2 diffractometer	2227 independent reflections
Radiation source: fine-focus sealed tube	1872 reflections with *I* > 2σ(*I*)
graphite	*R*_int_ = 0.038
Detector resolution: 13.6612 pixels mm^-1^	θ_max_ = 27.5°, θ_min_ = 3.4°
ω scan	*h* = −16→16
Absorption correction: multi-scan (*CrystalClear*; Rigaku, 2005)	*k* = −8→8
*T*_min_ = 0.732, *T*_max_ = 0.871	*l* = −16→16
9255 measured reflections	

### Refinement

**Table:**

Refinement on *F*^2^	Primary atom site location: structure-invariant direct methods
Least-squares matrix: full	Secondary atom site location: difference Fourier map
*R*[*F*^2^ > 2σ(*F*^2^)] = 0.034	Hydrogen site location: inferred from neighbouring sites
*wR*(*F*^2^) = 0.072	H-atom parameters constrained
*S* = 1.13	*w* = 1/[σ^2^(*F*_o_^2^) + (0.0216*P*)^2^ + 0.2674*P*] where *P* = (*F*_o_^2^ + 2*F*_c_^2^)/3
2227 reflections	(Δ/σ)_max_ < 0.001
115 parameters	Δρ_max_ = 0.30 e Å^−^^3^
4 restraints	Δρ_min_ = −0.35 e Å^−^^3^

### Special details

**Table:**

Geometry. All e.s.d.'s (except the e.s.d. in the dihedral angle between two l.s. planes) are estimated using the full covariance matrix. The cell e.s.d.'s are taken into account individually in the estimation of e.s.d.'s in distances, angles and torsion angles; correlations between e.s.d.'s in cell parameters are only used when they are defined by crystal symmetry. An approximate (isotropic) treatment of cell e.s.d.'s is used for estimating e.s.d.'s involving l.s. planes.
Refinement. Refinement of *F*^2^ against ALL reflections. The weighted *R*-factor *wR* and goodness of fit *S* are based on *F*^2^, conventional *R*-factors *R* are based on *F*, with *F* set to zero for negative *F*^2^. The threshold expression of *F*^2^ > σ(*F*^2^) is used only for calculating *R*-factors(gt) *etc*. and is not relevant to the choice of reflections for refinement. *R*-factors based on *F*^2^ are statistically about twice as large as those based on *F*, and *R*- factors based on ALL data will be even larger.

### Fractional atomic coordinates and isotropic or equivalent isotropic displacement parameters (Å^2^)

**Table:**

	*x*	*y*	*z*	*U*_iso_*/*U*_eq_	
Co1	0.0000	0.0000	0.5000	0.02435 (11)	
Cl1	0.62659 (4)	0.99536 (7)	0.26701 (4)	0.04265 (15)	
O1W	−0.10877 (11)	0.0642 (2)	0.33800 (10)	0.0348 (3)	
H1WA	−0.1770	0.0308	0.3213	0.052*	
H1WB	−0.1014	0.1829	0.3137	0.052*	
N2	0.26587 (14)	0.6024 (3)	0.55539 (14)	0.0406 (4)	
H2A	0.2560	0.4991	0.5944	0.061*	
H2B	0.2997	0.7116	0.5990	0.061*	
O2W	0.03539 (12)	−0.2770 (2)	0.44423 (12)	0.0469 (4)	
H2WA	0.1036	−0.3409	0.4710	0.070*	
H2WB	−0.0087	−0.3427	0.3854	0.070*	
C7	0.33808 (16)	0.3068 (3)	0.33515 (15)	0.0366 (4)	
H7	0.3225	0.1808	0.3001	0.044*	
C2	0.28925 (14)	0.3621 (3)	0.41754 (14)	0.0281 (4)	
C3	0.31432 (14)	0.5491 (3)	0.47396 (15)	0.0292 (4)	
N1	0.14159 (13)	0.1374 (2)	0.46523 (13)	0.0365 (4)	
C5	0.43102 (16)	0.6305 (4)	0.35985 (18)	0.0451 (5)	
H5	0.4774	0.7220	0.3393	0.054*	
C4	0.38513 (16)	0.6849 (3)	0.44191 (17)	0.0389 (5)	
H4	0.4012	0.8116	0.4760	0.047*	
C1	0.20887 (15)	0.2307 (3)	0.44319 (15)	0.0297 (4)	
C6	0.40921 (17)	0.4417 (4)	0.30739 (17)	0.0436 (5)	
H6	0.4424	0.4067	0.2539	0.052*	

### Atomic displacement parameters (Å^2^)

**Table:**

	*U*^11^	*U*^22^	*U*^33^	*U*^12^	*U*^13^	*U*^23^
Co1	0.02442 (18)	0.02295 (18)	0.02613 (18)	−0.00363 (13)	0.00896 (14)	−0.00016 (13)
Cl1	0.0385 (3)	0.0368 (3)	0.0443 (3)	−0.0048 (2)	0.0024 (2)	−0.0060 (2)
O1W	0.0330 (7)	0.0356 (7)	0.0326 (7)	−0.0032 (6)	0.0067 (6)	0.0048 (6)
N2	0.0416 (9)	0.0438 (10)	0.0384 (9)	−0.0046 (8)	0.0157 (8)	−0.0119 (8)
O2W	0.0411 (8)	0.0367 (8)	0.0517 (9)	0.0072 (6)	0.0001 (7)	−0.0150 (7)
C7	0.0305 (10)	0.0490 (12)	0.0309 (10)	0.0002 (9)	0.0110 (8)	−0.0040 (9)
C2	0.0214 (8)	0.0363 (10)	0.0270 (9)	−0.0044 (8)	0.0084 (7)	0.0020 (8)
C3	0.0222 (9)	0.0344 (10)	0.0284 (9)	0.0000 (7)	0.0049 (7)	0.0017 (7)
N1	0.0336 (8)	0.0413 (9)	0.0365 (9)	−0.0094 (8)	0.0139 (7)	0.0002 (7)
C5	0.0287 (10)	0.0601 (14)	0.0461 (12)	−0.0085 (10)	0.0117 (9)	0.0209 (11)
C4	0.0295 (10)	0.0354 (10)	0.0467 (12)	−0.0068 (8)	0.0058 (9)	0.0047 (9)
C1	0.0286 (9)	0.0333 (10)	0.0270 (9)	−0.0031 (8)	0.0089 (8)	−0.0022 (7)
C6	0.0325 (10)	0.0703 (15)	0.0325 (11)	0.0001 (10)	0.0167 (9)	0.0073 (10)

### Geometric parameters (Å, °)

**Table:**

Co1—O1W	2.0899 (14)	C7—C6	1.381 (3)
Co1—O1W^i^	2.0899 (14)	C7—C2	1.415 (2)
Co1—O2W	2.0550 (13)	C7—H7	0.9300
Co1—O2W^i^	2.0550 (13)	C2—C3	1.405 (3)
Co1—N1	2.1566 (15)	C2—C1	1.441 (2)
Co1—N1^i^	2.1566 (15)	C3—C4	1.408 (3)
O1W—H1WA	0.8377	N1—C1	1.147 (2)
O1W—H1WB	0.8551	C5—C4	1.385 (3)
N2—C3	1.398 (2)	C5—C6	1.392 (3)
N2—H2A	0.8715	C5—H5	0.9300
N2—H2B	0.9196	C4—H4	0.9300
O2W—H2WA	0.9097	C6—H6	0.9300
O2W—H2WB	0.8784		
			
O2W—Co1—O2W^i^	180.00 (8)	Co1—O2W—H2WB	125.5
O2W—Co1—O1W	89.38 (5)	H2WA—O2W—H2WB	109.6
O2W^i^—Co1—O1W	90.62 (5)	C6—C7—C2	119.31 (19)
O2W—Co1—O1W^i^	90.62 (5)	C6—C7—H7	120.3
O2W^i^—Co1—O1W^i^	89.38 (5)	C2—C7—H7	120.3
O1W—Co1—O1W^i^	180.00 (5)	C3—C2—C7	121.28 (16)
O2W—Co1—N1	91.13 (6)	C3—C2—C1	117.92 (15)
O2W^i^—Co1—N1	88.87 (6)	C7—C2—C1	120.75 (17)
O1W—Co1—N1	91.66 (6)	N2—C3—C2	120.91 (16)
O1W^i^—Co1—N1	88.34 (6)	N2—C3—C4	121.11 (17)
O2W—Co1—N1^i^	88.87 (6)	C2—C3—C4	117.90 (17)
O2W^i^—Co1—N1^i^	91.13 (6)	C1—N1—Co1	171.82 (16)
O1W—Co1—N1^i^	88.34 (6)	C4—C5—C6	121.43 (18)
O1W^i^—Co1—N1^i^	91.66 (6)	C4—C5—H5	119.3
N1—Co1—N1^i^	180.0	C6—C5—H5	119.3
Co1—O1W—H1WA	118.2	C5—C4—C3	120.27 (19)
Co1—O1W—H1WB	115.0	C5—C4—H4	119.9
H1WA—O1W—H1WB	111.8	C3—C4—H4	119.9
C3—N2—H2A	113.3	N1—C1—C2	175.48 (19)
C3—N2—H2B	114.3	C7—C6—C5	119.73 (18)
H2A—N2—H2B	113.3	C7—C6—H6	120.1
Co1—O2W—H2WA	124.4	C5—C6—H6	120.1

Symmetry codes: (i) −*x*, −*y*, −*z*+1.

### Hydrogen-bond geometry (Å, °)

**Table:**

*D*—H···*A*	*D*—H	H···*A*	*D*···*A*	*D*—H···*A*
O1W—H1WA···Cl1^ii^	0.84	2.33	3.1600 (16)	170
O1W—H1WB···Cl1^iii^	0.86	2.27	3.1099 (15)	167
O2W—H2WA···N2^iv^	0.91	1.99	2.868 (2)	162
O2W—H2WB···Cl1^v^	0.88	2.27	3.1438 (17)	178
N2—H2B···Cl1^vi^	0.92	2.53	3.4433 (18)	172

Symmetry codes: (ii) *x*−1, *y*−1, *z*; (iii) −*x*+1/2, *y*−1/2, −*z*+1/2; (iv) *x*, *y*−1, *z*; (v) −*x*+1/2, *y*−3/2, −*z*+1/2; (vi) −*x*+1, −*y*+2, −*z*+1.
